# Harm Reduction in Peer-Assisted Telemedicine for Hepatitis C: Secondary Outcomes of a Randomized Controlled Trial

**DOI:** 10.3390/v16091455

**Published:** 2024-09-13

**Authors:** Hunter Spencer, Devin Gregoire, Gillian Leichtling, Megan Herink, Andrew Seaman, P. Todd Korthuis, Ryan Cook

**Affiliations:** 1 Division of General Internal Medicine & Geriatrics, Oregon Health & Science University, Portland, OR 97239, USA; gregoird@ohsu.edu (D.G.); seaman@ohsu.edu (A.S.); korthuis@ohsu.edu (P.T.K.); cookry@ohsu.edu (R.C.); 2 Comagine Health, Portland, OR 97232, USA; gleichtling@comagine.org; 3 College of Pharmacy, Oregon State University, Corvallis, OR 97331, USA; herink@ohsu.edu; 4 Central City Concern, Portland, OR 97209, USA

**Keywords:** people who use drugs (PWUD), hepatitis C virus (HCV), harm reduction

## Abstract

Hepatitis C (HCV) treatment for people who use drugs (PWUD) decreases injection drug use and injection equipment sharing. We examined changes in injection drug use and injection equipment sharing following HCV treatment in a randomized trial comparing peer-assisted telemedicine for HCV treatment (TeleHCV) versus peer-assisted usual care in rural PWUD. We hypothesize that TeleHCV reduces risky behaviors and peers facilitate this change. We used mixed-effects logistic regression to describe participant-level (*n* = 203) associations between both injection drug use and injection equipment sharing and randomized groups, frequency of peer contact, HCV treatment initiation, HCV cure, and time. Risky behaviors were surveyed at baseline and 12 and 36 weeks after HCV treatment completion. Injection drug use declined more over time in TeleHCV participants vs. control at 12 weeks (adjusted odds ratio [aOR] = 0.42, 95% CI 0.20–0.87, *p* = 0.02) and 36 weeks (aOR = 0.48, 95% CI 0.21–1.08, *p* = 0.076). Injection drug use decreased more with a greater number of peer interactions, with reductions among participants in the 3rd quartile exceeding those in the 1st quartile of peer interactions at 12 weeks (aOR = 0.75, 95% CI 0.57–0.99, *p* = 0.04). Similarly, injection equipment sharing decreased over time, with reductions among participants in the 3rd quartile exceeding those in the 1st quartile of peer interactions at 36 weeks (aOR = 0.08, 95% CI 0.01–0.97, *p* = 0.047). Peer-assisted telemedicine for HCV treatment decreases injection drug use and injection equipment sharing; peers contribute to this effect.

## 1. Introduction

The hepatitis C virus (HCV) epidemic disproportionately affects people who use drugs (PWUD), with an estimated 38.8% of all PWUD globally experiencing current HCV infection [1]. The World Health Organization’s plan to eliminate HCV by 2030 identifies PWUD as a priority population and calls for increased utilization of both HCV treatment and harm reduction as key steps in HCV epidemic control [2]. Harm reduction refers to behaviors and interventions designed to mitigate the negative impacts of substance use [3], such as reductions in overdose and infectious complications of substance use and improved healthcare experiences for PWUD [4]. Harm reduction focused on HCV epidemic control includes interventions that decrease behaviors most associated with HCV transmission, such as injection drug use and injection equipment sharing [5]. Harm reduction interventions decrease the rate of HCV transmission [6]. A meta-analysis of 28 studies (*n* = 6279) in North America, Europe, China, and Australia found harm reduction interventions that combined medications for opioid use disorder (MOUD) and syringe exchange decreased HCV transmission nearly fourfold compared to groups without access to MOUD and either limited or absent access to syringe exchange (Relative Risk [RR] = 0.26, 95% CI = 0.07–0.89) [6].

Similarly, engaging in HCV treatment is associated with adoption of less risky drug use, including decreased injection frequency and decreased injection equipment sharing [7,8]. An analysis of two open-label observational studies of HCV treatment with direct-acting antivirals (DAA) conducted in Australia, North America, and Europe demonstrated small reductions in opioid injecting (odds ratio [OR] = 0.95, 95% CI = 0.92–0.99) and injection equipment sharing (OR = 0.87, 95% CI = 0.80–0.94) [7]. The Hepatitis C Real Option (HERO) pragmatic trial evaluating delivery systems for HCV treatment with DAA conducted in eight United States (U.S.) cities demonstrated decreases in any injection use (OR = 0.81, 95% CI = 0.78–0.84) and injection equipment sharing (OR = 0.88, 95% CI = 0.86–0.90) [8]. These risk reduction behaviors were more prevalent while participants were undergoing HCV treatment but persisted at 60 weeks. A study utilizing telemedicine administered in predominantly urban opioid treatment programs found decreases in opioid use disorder severity among participants who were cured of HCV [9]. Pairing HCV treatment with harm reduction services leverages the relationship between HCV treatment, risk reduction tools, and HCV transmission to form a more patient-centered solution to the HCV epidemic.

Rural PWUD in the U.S. face elevated barriers to both HCV treatment and harm reduction services, including decreased access, geographic distances, and stigma [10,11,12]. Consequently, little is known about the influence of HCV treatment on drug-related risk behaviors among rural PWUD. In a cross-sectional study of PWUD (*n* = 1982) in ten rural areas throughout the U.S., 72% of those with HCV antibody positivity reported at least daily injection drug use, and 74% reported sharing injection equipment [13]. Highly prevalent injection drug use and injection equipment sharing drives the HCV epidemic [14] and increases the risk for HCV re-infection after successful HCV treatment [15,16], presenting particular challenges in areas where access to harm reduction services is limited. In a cohort of PWUD using MOUD who were cured of HCV as part of a randomized trial, the incidence of reinfection was six times higher among those who continued to inject drugs (7.4 per 100 person-years) than the overall incidence of reinfection (1.22 per 100 person-years) [15]. In a cohort of PWUD cured of HCV (*n* = 448), those with ongoing risky opioid use or injection practices, defined by a hospitalization for complications of opioid use or injection, were at far higher risk of reinfection (adjusted hazard ratio = 12.9, 95%CI = 2.2–76.0) [16]. Despite the clear need for risk reduction interventions among rural PWUD, the effect of HCV treatment on harm reduction practices in rural U.S. populations remains unclear.

A recent pragmatic randomized controlled trial compared peer-assisted telemedicine HCV treatment (TeleHCV) versus peer-assisted referral to local providers among PWUD in seven rural Oregon counties [17,18]. The trial found large differences in HCV treatment initiation and viral clearance (primary outcome) in participants allocated to TeleHCV compared to peer-assisted referral to local providers [18]. TeleHCV is a packaged intervention consisting of both low-barrier access to telemedicine for HCV treatment and peer-based harm reduction. Peer-based harm reduction services, provided by “peers” with previous or ongoing substance use, improve harm reduction behaviors among PWUD in both urban [19,20] and rural areas [21]. However, the effect of pairing telemedicine HCV treatment with peer-based services on engagement in reducing drug-related risk behaviors is unknown. In this manuscript, we examine changes in risky behaviors over time, including injection substance use and injection equipment sharing. We hypothesize that allocation to the TeleHCV intervention versus allocation to peer-assisted referral to local providers reduces drug-related risk behaviors and that peers facilitate this change.

## 2. Materials and Methods

This paper describes analyses of secondary outcomes from all participants in the Oregon HOPE TeleHCV randomized controlled trial (NCT04798521), which is described fully elsewhere [17]. Briefly, people in seven rural Oregon counties with detectable HCV ribonucleic acid (RNA) > 15 IU/mL and self-reported past-90-day injection drug use or non-prescription opiate use were randomized to peer-assisted telemedicine for HCV treatment (TeleHCV) or peer-assisted referral to usual care with local providers (Enhanced usual care; EUC). Uninsured participants who were ineligible for Oregon Medicaid at enrollment were excluded. “Peers” in the study were people with lived experience of substance use and in recovery who were trained and certified by the state of Oregon as peer recovery support specialists. In both groups, peers provided medical care navigation, including assistance enrolling in Oregon Medicaid, and harm reduction services, including injection equipment supplies. As a pragmatic study, many procedures followed local protocols, including matching peers to clients, peer-led discussions regarding injection drug use and injection equipment sharing, and insurance coverage for HCV treatment. Peer-client matching varied by site, but usually peers met potential clients at syringe exchanges or harm reduction outreach events and formed peer-client relationships based on availability and mutual agreement. HCV treatment was paid for by participants’ insurance. Providers prescribed 8- or 12-week regimens of direct-acting antivirals at their clinical discretion. In both arms, peers facilitated phlebotomy for patients by providing transportation and co-attending phlebotomy visits and sharing lab results. In the TeleHCV arm, peers co-attended virtual visits while providing hardware to access the virtual visit meeting room (Zoom Video Communications Inc.: San Jose, CA, USA). In the EUC arm, peers facilitated hand-off to existing local peers to assist the EUC group with HCV care navigation. The primary outcome was HCV viral clearance 12 weeks post-treatment completion (sustained virologic response [SVR]12) or 32 weeks post-randomization in participants who never started treatment. Peers surveyed participants at baseline and two subsequent follow-up timepoints, which were determined by when or if participants started HCV treatment. Participants who started HCV treatment were surveyed at 12 weeks after treatment completion (SVR12) and 36 weeks after treatment completion (SVR36). For participants who never started treatment, the SVR12 timepoint was defined as 32 weeks post-randomization, and SVR36 was defined as 52 weeks post-randomization. Participants were offered cash incentives ranging from $15–$40 for lab draws and surveys. Peers in both arms worked to mitigate dropout throughout follow-up. The Oregon Health & Science University institutional review board approved the study protocol (STUDY00020911).

### 2.1. Outcomes

Participants were surveyed at baseline, SVR12, and SVR36 and self-reported the frequency with which they injected drugs during the last 30 days. Participants who reported injecting drugs were asked two additional questions regarding the preceding 30 days: (1) the number of times they had used a syringe or needle previously used by someone else, and (2) the number of times they had used a cotton, cooker, spoon, or water for rinsing or mixing that had been previously used by someone else. Both injection frequency and frequency of injection equipment sharing were dichotomized as “engaged” and “did not engage” in these behaviors. Non-injection routes of administration were not surveyed among those who did not report injection drug use in the last 30 days.

### 2.2. Exposures

Randomization group (TeleHCV vs. EUC), HCV treatment initiation (yes vs. no), and HCV cure (yes vs. no) were treated as binary measures. HCV treatment initiation was defined as a self-report of taking the first dose of HCV treatment and confirmation of medication receipt from the pharmacy. HCV cure was defined as a viral load <15 international units per milliliter at the SVR12 timepoint. The frequency of peer contact was defined by summing the number of instances in which a peer interacted with a participant to provide specific types of support or assistance. Peers in both arms recorded interactions with clients, which could be scheduled within study protocol (for example at the SVR12 and SVR36 timepoints) or initiated by either clients or peers. Peers counted any contact with participants in both arms as an interaction, and each interaction was categorized by the peer from a standardized list of interaction types. Types of peer support included goal setting and tracking, transportation, appointment attendance, harm reduction services, medication monitoring, physical and mental health discussions, and housing assistance, among other services. Each peer interaction with a participant was counted as a single interaction, even if multiple types of assistance were provided at the time. Because the duration of follow-up and peer engagement varied based on when or if a participant started HCV treatment, the frequency of peer contact was defined as the total number of peer contacts within the first 90 days after the baseline survey was administered. Peer contact frequency was analyzed as a linear measure and then described by the first and third quartiles of contacts to improve interpretability.

### 2.3. Statistical Analyses

Participant demographics, behaviors, and clinical characteristics were described with univariate statistics (means, standard deviations, frequencies, and percentages) and stratified by randomization group. Chi-square tests, *t*-tests, and Mann–Whitney U tests compared baseline characteristics of the groups in categorical, normal, and non-normal measures, respectively. Analyses of changes in HCV risk behaviors over time were estimated using mixed-effects logistic regression. Due to the large number of outcomes and exposures of interest, we employed a staged modeling strategy. For each exposure (randomized group, HCV treatment initiation, HCV cure, and frequency of peer interaction) and outcome (injection equipment sharing and injection drug use) combination, we first compared a model including main effects plus interactions between exposure and time (a categorical variable defined as baseline, SVR12, or SVR36) to a model with main effects only (i.e., no interactions) using a type III likelihood ratio (LR) test. The significance of the LR test indicated differences in change in outcome over time by exposure status, prompting further comparisons between exposure groups at each timepoint. A non-significant LR test suggested that changes over time were similar between exposure groups, and no further action was taken.

All models were adjusted for baseline age, gender, race, past 6-month homelessness, and substance use severity, defined as the number of days in the past 30 each participant reported using a substance to get high (range of 0–30; substances included heroin, fentanyl, prescription full-agonist opioids, buprenorphine, prescription anxiety drugs, methamphetamine, gabapentin, and kratom). Models also included participant-level random intercepts to account for repeated measurements; given the mixed models, missing data were treated as missing at random. Estimated marginal means were used to generate timepoint and exposure group-specific estimates of predicted outcome levels, and comparisons between groups at each time were contrasts of such means. Statistical analyses were conducted in R (version 4.3.1) with use of the ‘lme4’, ‘emmeans’, and ‘ggplot2′ packages. All statistical tests performed were two-sided with a significance level of 0.05.

## 3. Results

### 3.1. Participants

Participants (*n* = 203) averaged 41.6 years of age (SD 11.2) and were predominately male (62%) and White (88%) (Table 1). Nearly three-quarters (74%) had a high school equivalent education or less, and 70% had experienced homelessness in the past six months. At baseline, one participant had biochemical evidence of hepatic fibrosis, defined as a Fibrosis 4 score (Fib-4) > 3.25, and 1 participant had decompensated cirrhosis, defined as a Child–Pugh score ≥7. The median number of days of substance use within the last 30 days was 30 (IQR 15–30), with 88% and 62% of participants using methamphetamine and opioids within the last 30 days, respectively, and 58% of participants using both opioids and methamphetamine. The median number of peer contacts in the first 90 days following baseline was 1 (IQR 1–4). Half of the peer contacts occurred in person (49%), with the most common reasons for contact being physical health discussion (45%), goal creation or progress update (45%), starting HCV medication (34%), provision of harm reduction services (29%), and co-attendance of medical appointments (29%). One hundred ninety participants (93.5%) were HCV treatment naïve at baseline; about half of participants initiated HCV treatment during the trial (48%), and 39% of participants were cured of HCV at SVR36. In the EUC group (*n* = 103), 13% initiated HCV treatment, and 16% were cured of HCV at SVR36. In the TeleHCV group (*n* = 100), 85% initiated HCV treatment, and 63% were cured at SVR36. At baseline, TeleHCV participants were more likely to have used opioids in the past 30 days (*p* = 0.03). The number of participants who responded to follow-up surveys decreased over time, from 203 at baseline to 171 and 129 at SVR12 and SVR36, respectively.

### 3.2. Injection Drug Use

**Figure 1:**
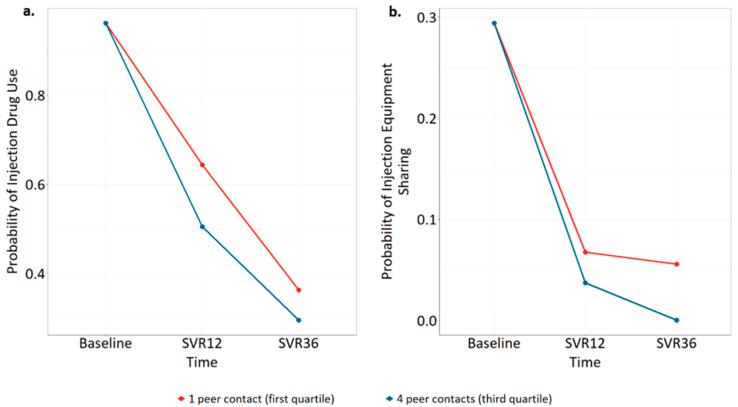
Model-predicted changes in (**a**) injection drug use by frequency of peer contact and (**b**) injection equipment sharing by frequency of peer contact.

Injection drug use within the last 30 days was highly prevalent at baseline, with 167 participants (82%) reporting injection drug use, declining to 103 (60%) and 65 (50%) at SVR12 and SVR36. The type III likelihood ratio tests showed statistically significant interactions between time and randomized group (*p* = 0.009) and time and frequency of peer contact (*p* = 0.03). Conversely, there was little evidence of changes in risk behaviors by HCV treatment initiation status (type III test of interaction terms, *p* = 0.13) or HCV cure status (*p* = 0.20). Table 2 shows timepoint specific comparisons for variables with a significant type III test. Relative to the EUC group, participants in the TeleHCV arm were less likely to report injection drug use at SVR12 (adjusted OR = 0.42, 95% CI 0.20–0.87, *p* = 0.02) and SVR36 (aOR = 0.48, 95% CI 0.21–1.08, *p* = 0.08), although the latter comparison did not reach statistical significance. Additionally, adjusted models indicate that greater numbers of peer interactions were associated with a decrease in injection drug use at SVR12 (aOR third quartile of peer contacts vs. first = 0.75, 95% CI 0.57–0.99, *p* = 0.04). Figure 1a shows the model-predicted changes in injection drug use over time by the first and third quartiles of peer contact exposures.

### 3.3. Injection Equipment Sharing

Among those who injected drugs in the last 30 days, 56 participants reported injection equipment sharing at baseline (34%), falling to 9 at SVR12 (8.7%) and 6 at SVR36 (9.2%). The type III test showed statistically significant interactions between time and frequency of peer contact (*p* = 0.001) but not time and randomized group (*p* = 0.41) or time and HCV treatment initiation status (*p* = 0.29). The type III test of time and HCV cure status failed to converge because zero participants who were cured of HCV reported sharing injection equipment at SVR36. Thus, an interaction between time and HCV cure could not be investigated further. Table 2 shows timepoint-specific comparisons, demonstrating that greater frequency of peer interactions was associated with decreased injection equipment sharing at SVR36 (aOR third quartile of peer contacts vs. first = 0.08, 95% CI 0.01–0.97, *p* = 0.047). Figure 1b displays the model-predicted decline in injection equipment sharing over time for participants by the first and third quartiles of peer interaction.

## 4. Discussion

Overall, results suggest that peer-assisted telemedicine for HCV is effective at reducing drug-related risk behaviors among rural PWUD. Risky behaviors decreased over time, but there was no additional decrease in risky behaviors among those who initiated HCV treatment and those who did not. In contrast, injection drug use significantly declined in the TeleHCV group compared to the EUC group, and the benefit persisted to 36 weeks after HCV treatment completion. Furthermore, greater intensity of peer interactions was associated with a decline in injection drug use and equipment sharing, based on the inverse association between peer-contact frequency and these risky behaviors. This suggests that the entire packaged TeleHCV intervention, highlighted by peer-based services, drove behavior change more strongly than HCV treatment initiation alone. Peer intervention in both arms of this trial likely explains why both groups reduced drug-related risk behaviors over time, despite large differences in HCV treatment and cure between randomized groups [18,22].

Participants in this study decreased injection drug use more dramatically compared to other studies of harm reduction during HCV treatment with both DAA [7,8] and older regimens [23]. The involvement of peers in this trial is a unique feature that likely explains greater improvements. The HERO study, which included patient navigators and some peers in one intervention arm, demonstrated a larger effect size [8] than non-randomized DAA implementation studies, which did not include peers [7]. Unlike other studies [8,9], we analyzed drug-related risk behaviors among all participants—those who initiated HCV treatment and were cured and those who did not. While most participants in the EUC group did not initiate HCV treatment or reach cure, injection frequency still declined. Our data suggest that peer involvement in both study arms accounts for this finding. Notably, the decline in injection drug use occurred within a sample characterized by frequent use of both opioids and stimulants. In a recent cross-sectional study of rural PWUD with HCV antibody positivity, use of amphetamines and opioids, either separately or co-injected, was associated with increased injection frequency compared to either amphetamine or opioid use alone, making our findings in this high-risk population all the more striking [13].

In contrast to the present study, other trials have demonstrated an effect of HCV treatment on decreasing injection equipment sharing [7]. Here, peer contact was a better predictor of decreased injection equipment sharing than randomized group, HCV treatment, or HCV cure status. This highlights the impact of the active control group in this study, in which peer services were provided to both arms and continued to offer harm reduction services regardless of HCV treatment initiation, which may have attenuated the influence of both the telemedicine intervention and HCV treatment. This finding adds to the literature supporting the role of peers as harm reduction providers for PWUD. Qualitative research demonstrates that peer-based harm reduction is feasible and acceptable to PWUD [21,24,25] and non-randomized program evaluations have described their impact [26], but this study quantifies the role of peers in promoting harm reduction in the setting of HCV treatment.

An additional strength of this study is the inclusion of rural PWUD with a high proportion experiencing houselessness. This population is often excluded from clinical trials but should be prioritized in HCV elimination programs. Given these sample characteristics, study retention was high over an extended period. Another strength is our modeling approach, which allows for a nuanced understanding of the differences that occurred over time and between groups. Our staged modeling approach decreased the total number of statistical comparisons and thus limited the likelihood of inflation bias [27]. Importantly, this study contributes new knowledge about the effect of harm reduction from a peer-assisted telemedicine intervention.

The limitations of the study include its nature as an analysis of secondary outcomes, which the main study was not powered to address. This probably predisposed our analysis to a type II error, given the improvement in harm reduction behaviors in both groups over time and the overlapping exposures to peers between the intervention and control. Additionally, only the treatment group was randomized, and analyses of treatment initiation, HCV cure, and intensity of peer interactions are subject to confounding, despite controlling for several factors to mitigate this issue. Another limitation is that participants self-reported risky behaviors “during the last 30 days” even though the follow-up intervals were longer than 30 days; thus, risky behaviors may be underreported. Generalizability beyond rural U.S. settings may be limited given highly variable access to harm reduction for PWUD globally [28]. Because we recruited from rural communities, generalizability to urban settings is also limited. The effect of a peer-facilitated telemedicine HCV intervention could be less significant in areas with greater population density or more accessible harm reduction. Few other studies assess changes in harm reduction due to telemedicine HCV treatment, but one study that enrolled 15% rural people found decreases in substance use disorder severity [9]. Unlike other studies, we recruited PWUD rather than restricting recruitment to people who inject drugs (PWID) [8]. However, the current study had a high proportion of PWID at baseline, and the relative decline in risky injection behaviors was similar in an urban cohort composed entirely of PWID [8]. Finally, while retention until follow-up was relatively high (84% at the SVR12 timepoint and 63% at the SVR36 timepoint), the sample retained to follow-up may not represent the entire OR-HOPE study sample.

## 5. Conclusions

In this study, peer-assisted telemedicine for HCV treatment decreased injection drug use and injection equipment sharing. HCV treatment initiation alone was not sufficient to reduce risky behaviors, but greater intensity of peer interactions predicted a greater reduction in injection drug use and decreased injection equipment sharing. These results support the role of peers in facilitating HCV treatment and integrating peer-delivered harm reduction services. Peer-assisted telemedicine for HCV treatment responds to international calls to increase access for both HCV treatment and risk reduction tools among rural PWUD, a priority population for HCV epidemic control. Scaling this intervention for other rural areas and incorporating peers more broadly into HCV treatment are important next steps.

## Figures and Tables

**Table 1:** Demographic characteristics of study participants.

	Overall (N = 203)	EUC (N = 103)	TeleHCV (N = 100)	*p*
Age ^1^	41 (33, 50)	41 (34, 53)	41 (32, 49)	0.37
Male	126 (62.1)	68 (66.0)	58 (58.0)	0.30
Race				0.38
American Indian	14 (6.9)	9 (8.7)	5 (5.0)	
Mixed race	5 (2.5)	3 (2.9)	2 (2.0)	
Other	5 (2.5)	1 (1.0)	4 (4.0)	
White	179 (88.2)	90 (87.4)	89 (89.0)	
Hispanic	11 (5.4)	7 (6.8)	4 (4.0)	0.57
Education				0.79
Less than high school	57 (28.1)	29 (28.2)	28 (28.0)	
High school/GED	93 (45.8)	47 (45.6)	46 (46.0)	
Some postsecondary	52 (25.6)	27 (26.2)	25 (25.0)	
Bachelor’s or higher	1 (0.5)	0 (0.0)	1 (1.0)	
Experienced homelessness in past 6 months	141 (69.5)	74 (71.8)	67 (67.0)	0.55
Frequency of substance use in past 30 days ^1^	30 [15, 30]	30 [11, 30]	30 [20, 30]	0.84
Used methamphetamine in past 30 days	179 (88.2)	89 (86.4)	90 (90.0)	0.57
Used opioids in past 30 days	126 (62.1)	56 (54.4)	70 (70.0)	0.03
Injected drugs in past 30 days	167 (82.3)	80 (77.7)	87 (87.0)	0.12
Shared injection equipment in past 30 days	56 (33.7)	27 (33.8)	29 (33.7)	0.99
Number of peer contacts within 90 days of baseline ^1,2^	1 (1, 4)	1 (0, 1)	3 (1, 6)	<0.01
Initiated HCV treatment ^2^	98 (48.3)	13 (12.6)	85 (85.0)	<0.01
Cured of HCV at SVR36 ^2^	79 (38.9)	16 (15.5)	63 (63.0)	<0.01

^1^ Median (first and third quartiles). ^2^ Non-randomized measures.

**Table 2:** Effects of time by exposure interactions on injection drug use and injection equipment sharing.

	Injection Drug Use ^a^		Injection Equipment Sharing ^a^	
	Adjusted OR (95% CI)	*p*-Value	Adjusted OR (95% CI)	*p*-Value
SVR12 Timepoint				
Randomization group: TeleHCV vs. EUC	0.42 (0.20–0.87)	0.02	-	-
Frequency of peer contact, count ^b^	0.75 (0.57–0.99)	0.04	0.73 (0.41–1.31)	0.29
SVR36 Timepoint				
Randomization group: TeleHCV vs. EUC	0.48 (0.21–1.08)	0.076	-	-
Frequency of peer contact, count ^b^	0.86 (0.62–1.19)	0.36	0.08 (0.01–0.97)	0.047

^a^ Models adjusted for age, gender, race, past-6-month homelessness, and frequency of substance use in past 30 days; ^b^ Contrast compares the third quartile of peer contacts (4) to the first quartile (1).

## Data Availability

De-identified data are available from the authors upon request.
